# Fatal Refractory Radiation-Induced Biliary Stenosis (RIBS) and Liver Abscess Following Carbon-Ion Radiotherapy for Hepatocellular Carcinoma: A Case Report

**DOI:** 10.7759/cureus.104102

**Published:** 2026-02-23

**Authors:** Hideo Kidogawa, Takahito Tagami, Takatomo Yamayoshi, Junya Noguchi, Kohji Okamoto

**Affiliations:** 1 Department of Surgery, Kitakyushu City Yahata Hospital, Kitakyushu, JPN

**Keywords:** biliary stricture, carbon-ion radiotherapy, hepatocellular carcinoma, late toxicity, liver abscess, opportunistic infection, radiation-induced biliary stenosis, raoultella ornithinolytica

## Abstract

Carbon-ion radiotherapy (C-ion RT) is an effective modality for hepatocellular carcinoma (HCC); however, it carries a risk of radiation-induced biliary stenosis (RIBS), particularly for centrally located tumors. We report a rare but potentially fatal case of refractory RIBS following C-ion RT. A patient underwent C-ion RT for HCC located near the hepatic hilum. During long-term follow-up, despite no evidence of local tumor recurrence, the patient developed severe biliary stenosis. Due to the ischemic and rigid nature of RIBS, the stricture was highly resistant to standard endoscopic balloon dilation and repeated plastic stenting. Metallic stents were avoided to prevent complications associated with benign strictures, and surgical or percutaneous interventions were contraindicated due to the patient's poor baseline hepatic reserve and lobar atrophy. The clinical course was complicated by recurrent cholangitis, ultimately culminating in a fatal liver abscess and septic shock driven by the opportunistic pathogen *Raoultella ornithinolytica*. Because detailed dosimetric parameters were unavailable, definitive causality cannot be established; thus, C-ion RT is considered a significant contributory risk factor. RIBS is a rare but catastrophic late complication of C-ion RT for central HCC. The profound ischemic changes make standard endoscopic management highly challenging, predisposing patients to severe opportunistic infections. This case highlights the necessity of long-term vigilance, risk-adapted patient selection, and cautious multidisciplinary management.

## Introduction

Hepatocellular carcinoma (HCC) remains a leading cause of cancer-related mortality worldwide. While various treatment modalities exist, carbon-ion radiotherapy (C-ion RT) has emerged as a highly effective, non-invasive option [1,2,3]. However, because C-ion RT facilities require massive infrastructure, this promising modality is currently limited globally and predominantly concentrated in Japan and other parts of Asia [1,4].

Clinicians achieve excellent safety profiles and high local control rates when treating peripheral liver tumors with C-ion RT [2]. In stark contrast, managing centrally located HCCs near the hepatic hilum introduces significant risks to the central hepatobiliary tract, as high radiation doses can cause severe collateral damage to adjacent critical structures, including radiation-induced liver damage [5].

Consequently, radiation-induced biliary stenosis (RIBS) has surfaced as a severe late complication for central tumors. A recent retrospective study by Maki et al. [6] evaluated this risk, explicitly demonstrating that patients receiving high biological effective doses for tumors adjacent to the hepatic hilum face an elevated incidence (>10-15%) of late biliary strictures.

To further elucidate the natural history of this refractory complication, we report a rare case of a patient who developed severe RIBS following C-ion RT for a central HCC. This case specifically highlights the prolonged latency period of the stricture and the subsequent fatal infectious cascade driven by emerging opportunistic pathogens, thereby distinguishing this report from existing literature and emphasizing the critical need for long-term vigilance.

## Case presentation

**Figure 1:**
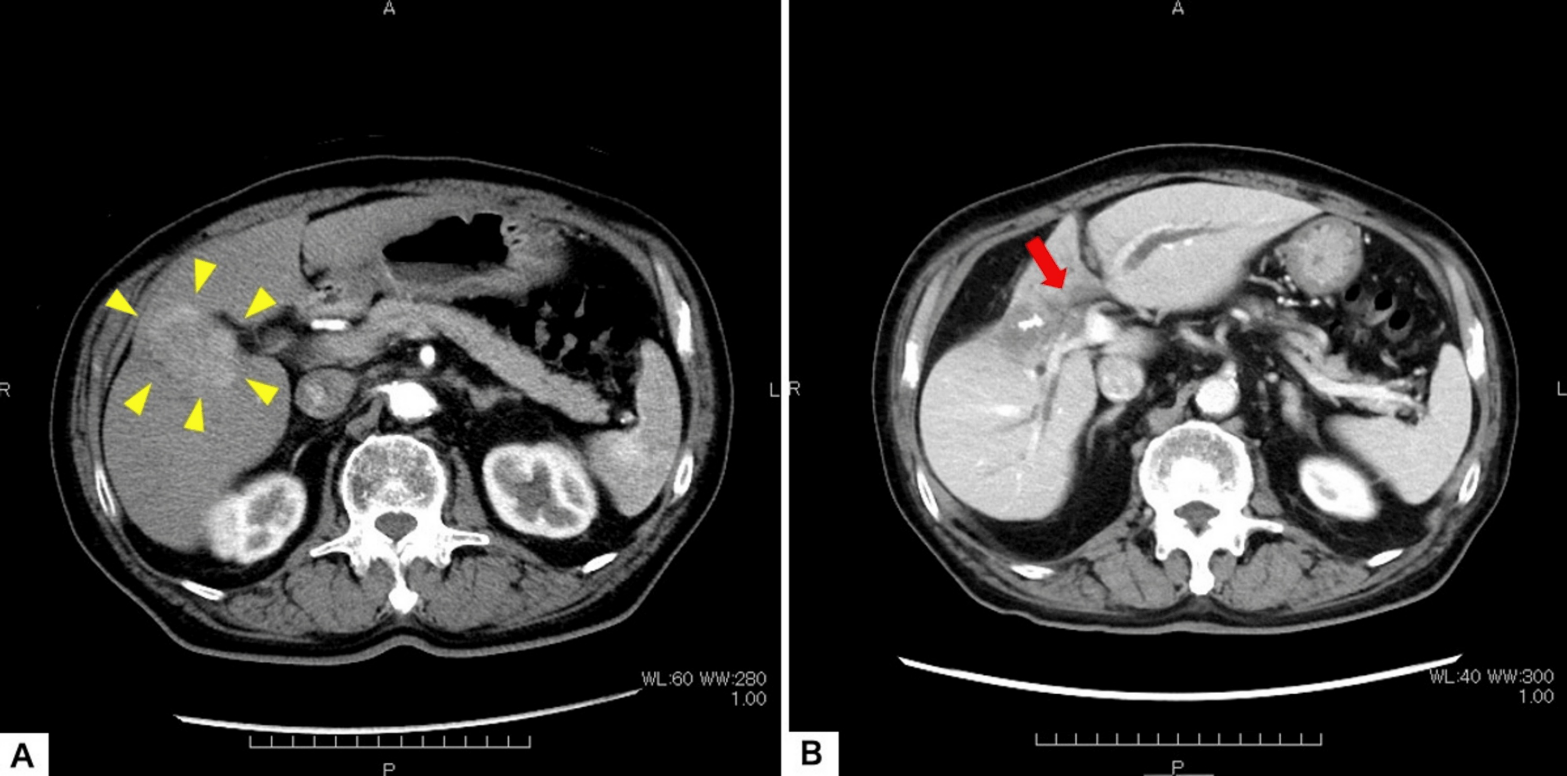
Radiographic evolution of radiation-induced biliary stenosis (RIBS). (A) Pre-treatment contrast-enhanced computed tomography (CT) (three months prior to carbon-ion radiotherapy (C-ion RT)) showing the viable hepatocellular carcinoma (HCC) in Segment 4/5 (arrowheads). (B) CT at the onset of jaundice (2.5 years post-C-ion RT) showing marked atrophy of S5 (arrow) and significant upstream biliary dilation (arrow).

A male in his 70s with a history of chronic hepatitis B (HBeAg seroconverted), type 2 diabetes mellitus, and paroxysmal atrial fibrillation presented with recurrent HCC. Six months prior to referral, he underwent transarterial chemoembolization (TACE) for tumors in liver segments 4 and 5 (S4/5). A follow-up computed tomography (CT) scan three months later showed residual viable tumor (Figure 1a). Due to his comorbidities and refusal of surgery, he was referred for particle therapy.

Prior to C-ion RT, the patient’s hepatic reserve was classified as Child-Pugh class A (score 5) and ALBI grade 1. Furthermore, the absence of esophageal varices or splenomegaly on computed tomography confirmed that no significant baseline portal hypertension was present. The patient underwent C-ion RT targeting the residual tumor in S4/5, with a treatment plan delivering a total dose of 60 GyE in four fractions. However, detailed dosimetric parameters, specifically the biliary maximum dose (Dmax), biological effective dose (BED) to the central hepatobiliary tract, and specific beam arrangements, were not available for analysis due to the referral nature of the case. He completed the treatment without acute complications. At the initial follow-up visit to our institution two months post-treatment, laboratory tests showed elevated hepatobiliary enzymes (AST 93 U/L, ALP 549 U/L, gamma-glutamyltransferase [GGT] 1015 U/L), likely reflecting background liver injury and recent therapy, but total bilirubin was within normal limits (1.1 mg/dL) (Table 1).

**Table 1:** Progression of laboratory findings and key clinical inflection points from initial referral to the terminal phase. Carbon-ion radiotherapy (C-ion RT) was completed prior to the initial visit. Key clinical inflection points, including the onset of radiation-induced biliary stenosis (RIBS), the first endoscopic retrograde cholangiopancreatography (ERCP), and liver abscess formation, are explicitly annotated in the column headers to correlate with the laboratory trends. Alb, albumin; AST, aspartate aminotransferase; ALT, alanine aminotransferase; ALP, alkaline phosphatase; GGT, gamma-glutamyltransferase; CRP, C-reactive protein; WBC, white blood cells; PT-INR, prothrombin time-international normalized ratio

Parameter	Reference range	2 months	29 months	30 months	31 months	35 months
Clinical event		Initial Visit	Onset of RIBS	First ERCP	Liver Abscess	Septic Shock
Alb (g/dL)	4.1-5.1	4.2	3.5	2.7	1.8	2
Total bilirubin (mg/dL)	0.4-1.5	1.1	6.2	23.3	4.3	2.7
AST (U/L)	13-30	93	83	37	133	42
ALT (U/L)	10-42	90	61	22	55	20
ALP (U/L)	38-113	549	737	357	600	515
γ-GTP (U/L)	13-64	1015	2459	831	678	207
CRP (mg/dL)	0.00-0.14	0.74	1.66	23.02	28.27	14.22
WBC (10^3/μL)	3.3-8.6	6.3	4.6	9.5	25.5	20.7
PT-INR	0.90-1.10	-	-	-	1.49	1.51

Approximately 2.5 years (29 months) post-treatment, the patient presented with overt jaundice. Laboratory findings revealed a significant progression of hepatobiliary dysfunction compared to the baseline visit, with Total Bilirubin rising to 6.2 mg/dL and γ-GTP peaking at 2459 U/L. Contrast-enhanced CT revealed marked atrophy of the irradiated Segment 5 parenchyma with calcification and significant upstream dilation of the intrahepatic bile ducts (Figure 1b). There was no evidence of local tumor recurrence, leading to a diagnosis of RIBS.

**Figure 2:**
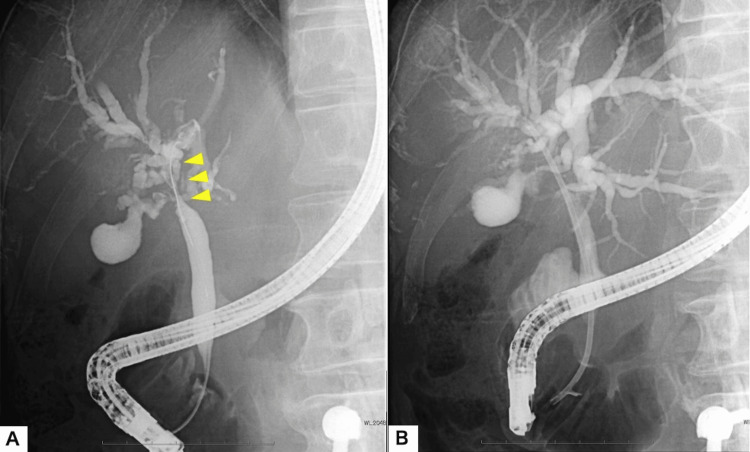
Endoscopic findings. (A) Endoscopic retrograde cholangiopancreatography (ERCP) cholangiogram revealing a severe, filiform stricture at the hepatic hilum (arrowheads), characteristic of ischemic radiation injury. (B) ERCP image obtained immediately after stent placement, showing successful deployment of the biliary stent across the stricture.

The patient required hospital admission one month after the onset of symptoms. Endoscopic Retrograde Cholangiopancreatography (ERCP) showed a severe, filiform stricture at the hepatic hilum (Figure 2a). Plastic stent placement and balloon dilation were performed. Although percutaneous transhepatic biliary drainage (PTBD) and surgical reconstruction were considered, they were deemed contraindicated due to the multiple, diffuse nature of the intrahepatic strictures, lobar atrophy, and the patient's poor hepatic reserve (Albumin 3.5 g/dL, PT-INR 1.05). Despite endoscopic intervention, jaundice rapidly worsened (T-Bil peaked at 23.3 mg/dL). Follow-up imaging confirmed the stent in situ, but a persistent stricture (Figure 2b).

**Figure 3:**
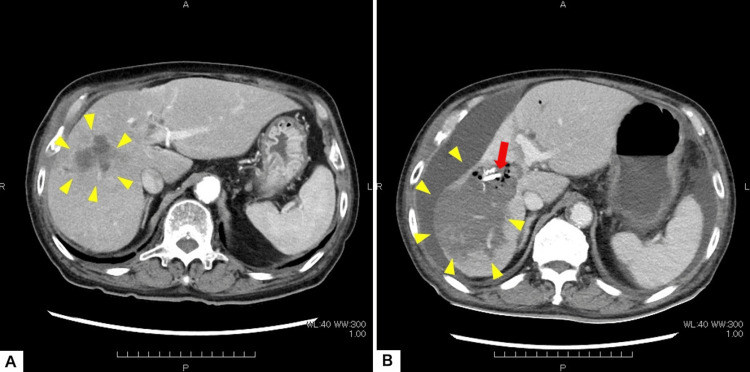
Progression of infectious complications. (A) Computed tomography (CT) showing initial formation of a liver abscess in the left lobe (arrowheads). (B) Terminal CT showing massive expansion of the abscess (arrowheads) despite drainage with a biliary stent (arrow).

Two months after the onset, he developed a liver abscess in the left lobe. Bile cultures grew *Streptococcus anginosus* and *Enterococcus faecalis*. Although antibiotics (Cefozopran) and drainage temporarily improved his condition, the stricture remained refractory, necessitating repeated stent exchanges (Figure 3a).

Six months after the onset of jaundice, the patient presented with septic shock and hepatic failure (T-Bil 2.7 mg/dL, PT-INR 1.33). Blood cultures were positive for *Raoultella ornithinolytica* and *Enterococcus faecalis*. Despite intensive care, the liver abscess expanded massively, and the patient died from liver failure shortly thereafter (Figure 3b).

## Discussion

This case illustrates the rare but potentially fatal nature of RIBS following C-ion RT. Drawing from this specific clinical experience, we discuss three critical aspects: anatomical risk factors, the underlying ischemic mechanism, and the impact of opportunistic infectious complications.

The occurrence of RIBS in this patient aligns with risk factors identified in recent studies. However, we must explicitly acknowledge the lack of detailed dose-volume histogram (DVH) parameters as a critical limitation. This absence of specific biliary dose metrics significantly weakens our ability to establish a definitive causal link between the radiation dose and the stricture, making it difficult to entirely exclude natural disease progression or background liver disease. Therefore, rather than a definitive cause, C-ion RT likely represents a significant contributory risk factor. Nevertheless, clinicians recognize the hepatic hilum as a high-risk zone; Maki et al. reported a 33.3% incidence of biliary stricture in the "Portal vein trunk branch area" within their cohort of 103 patients [6]. Furthermore, the J-CROS multi-institutional study by Kasuya et al. emphasized that the biliary tract acts as an organ at risk where high-dose irradiation causes irreversible damage [7].

Unlike malignant or inflammatory obstructions, researchers attribute the pathogenesis of RIBS to radiation-induced endothelial damage, microvascular ischemia, and subsequent fibrosis [8]. We explicitly contrast this ischemic mechanism with typical biliary strictures: the profound ischemia renders the stricture highly rigid, fibrotic, and resistant to standard endoscopic balloon dilation and repeated plastic stenting. We did not pursue metallic stents, as the gastroenterology literature highlights significant long-term complications associated with their use in benign strictures. Furthermore, the diffuse nature of the strictures and significant liver atrophy precluded salvage options such as percutaneous transhepatic biliary drainage (PTBD) or surgery. This refractory clinical course mirrors the severe management difficulties described in long-term survivor reports by Ohtaka et al. [9].

The terminal event in this case was precipitated by *Raoultella ornithinolytica* sepsis. While Righini et al. highlight this organism as an emerging opportunistic threat in immunocompromised patients [10], we must avoid overstating its epidemiological significance from a single case. Repeated biliary instrumentation, chronic obstruction, and ischemic hepatic necrosis likely served as the primary drivers of this fatal cascade, with the specific pathogen selection representing a secondary, opportunistic consequence.

Recent reviews and risk stratification studies emphasize that late biliary toxicity after C-ion RT is not merely incidental but a predictable risk, particularly in centrally located HCCs with high-dose exposure [11, 12]. Based on this experience and the anatomical risk stratification by Maki et al. [6], we cautiously offer the following hypothesis-generating suggestions and expert opinions, rather than prescriptive clinical directives:

Pre-treatment risk assessment

Clinicians should consider tumors in the hilar region as conferring a heightened risk of RIBS.

Long-term surveillance

We suggest extending the monitoring of biliary enzymes beyond the typical two-year window for patients with centrally irradiated tumors.

Empirical antimicrobial coverage: In patients with refractory RIBS undergoing repeated instrumentation, clinicians might consider empirical coverage for emerging opportunistic pathogens (including *Raoultella* species), strictly guided by local antibiograms and the principles of antimicrobial stewardship.

## Conclusions

Biliary stricture represents a rare but potentially fatal late complication of C-ion RT for centrally located HCCs. The profound ischemic nature of the stenosis renders it highly resistant to standard endoscopic interventions, creating a vulnerability to severe opportunistic infections and fatal cascades. While definitive causality requires further dosimetric studies, clinicians must maintain long-term vigilance, consider risk-adapted patient selection, and prioritize cautious multidisciplinary management for this refractory condition.

## References

[1] Byun HK, Kim C, Seong J (2023). Carbon ion radiotherapy in the treatment of hepatocellular carcinoma. Clin Mol Hepatol.

[2] Shibuya K, Katoh H, Koyama Y (2022). Efficacy and safety of 4 fractions of carbon-ion radiation therapy for hepatocellular carcinoma: a prospective study. Liver Cancer.

[3] Kaneko T, Makishima H, Wakatsuki M (2024). Carbon-ion radiotherapy for hepatocellular carcinoma with major vascular invasion: a retrospective cohort study. BMC Cancer.

[4] Schlaff CD, Krauze A, Belard A, O'Connell JJ, Camphausen KA (2014). Bringing the heavy: carbon ion therapy in the radiobiological and clinical context. Radiat Oncol.

[5] Hayashi K, Suzuki O, Wakisaka Y (2024). Prognostic analysis of radiation-induced liver damage following carbon-ion radiotherapy for hepatocellular carcinoma. Radiat Oncol.

[6] Maki K, Haga H, Katsumi T (2025). Adverse events after carbon-ion radiotherapy (CIRT) for hepatocellular carcinoma and risk factors for biliary stricture after CIRT: a retrospective study. Cancers (Basel).

[7] Kasuya G, Terashima K, Shibuya K (2019). Carbon-ion radiotherapy for cholangiocarcinoma: a multi-institutional study by and the Japan carbon-ion radiation oncology study group (J-CROS). Oncotarget.

[8] Zhu W, Zhang X, Yu M, Lin B, Yu C (2021). Radiation-induced liver injury and hepatocyte senescence. Cell Death Discov.

[9] Ohtaka T, Shiba S, Shibuya K (2022). Long-term survivor of hepatocellular carcinoma treated with repeated carbon ion radiotherapy and transarterial chemoembolization: a case report. Clin J Gastroenterol.

[10] Righini M, Titone M, Martelli D (2025). Bloodstream infection caused by Raoultella ornithinolytica in a chronic hemodialysis patient. Kidney Dial.

[11] Sasaki-Tanaka R, Abe H, Yoshida T (2025). Carbon-ion radiotherapy for hepatocellular carcinoma: current status and future prospects: a narrative review. J Clin Med.

[12] Hayashi K, Suzuki O, Ichise K (2025). A novel method for prognostic risk classification after carbon-ion radiotherapy for hepatocellular carcinoma using data-mining methods. Cancer Sci.

